# Development of hepatocellular carcinoma organoid model recapitulating HIF-1A metabolic signature

**DOI:** 10.1007/s10238-024-01521-x

**Published:** 2024-11-21

**Authors:** Mennatallah A. Khedr, Zainab Mohamed, Azza M. El-Derby, Malak M. Soliman, Amira Abdel Fattah Edris, Eman Badr, Nagwa El-Badri

**Affiliations:** 1 Center of Excellence for Stem Cells and Regenerative Medicine, Zewail City of Science and Technology, Giza, 12578 Egypt; 2 University of Science and Technology, Zewail City of Science and Technology, Giza, 12578 Egypt; 3 Bioinformatics Group, Center for Informatics Science (CIS), School of Information Technology and Computer Science, Nile University, Giza, 12588 Egypt; 4 Department of Pediatrics, Cairo University, Cairo, 11956 Egypt; 5 Faculty of Medicine, Kasr Al Ainy, Cairo University, Giza, 3240020 Egypt; 6 Faculty of Computers and Artificial Intelligence, Cairo University, Giza, 12613 Egypt

**Keywords:** Hypoxia, HIF-1A, Hepatocellular carcinoma, Organoids, Glycolysis, Metabolism

## Abstract

Hypoxia is one of the main hallmarks of hepatocellular carcinoma (HCC) resulting from improper oxygenation and insufficient nourishment of the HCC microenvironment. The effect of hypoxia is mediated by hypoxia-inducible factor-1A (HIF-1A) via targeting various downstream pathways, including glycolysis, angiogenesis, and survival signaling. However, HCC cell lines in a 2-dimensional (2D) setting do not resemble the metabolic signature of HCC. Here we aim to overcome these limitations by developing an HCC organoid that recapitulates the HIF-1A metabolic shift. The enrichment analysis of the RNA-Seq data revealed that HIF-1A-driven glycolytic shift is of the significant pathways. The established organoid model, using *xeno*-free plasma-derived extracellular matrix (ECM) as a scaffold and nutritive biomatrix, maintained its structural integrity and viability for up to 14 days; the comparative analysis of the cobalt (II) chloride (CoCl_2_)-treated organoids to the untreated ones unveiled reduced size and proliferative capacity. Interestingly, our organoid model showed an elevated expression of HIF-1A and glycolysis enzymes compared to their counterparts in the CoCl_2_-treated organoids. HIF-1A molecular expression-translated biochemical signature is further assessed in our spontaneously growing organoids showing an increase in glucose uptake, intracellular pyruvate, extracellular lactate dehydrogenase expression, and extracellular lactate production, while hydrogen peroxide (H_2_O_2_), a marker for oxidative metabolism, is reduced. Our data confirmed the potency of the established organoid model to mimic the molecular and biochemical HIF-1A-driven metabolism, which validates its potential use as an in vitro HCC model. Our model naturally simulates hypoxic conditions and simultaneous HIF-1A-dependent glycolysis within HCC rather than using of CoCl_2_-induced hypoxic conditions.

**Supplementary Information:**

The online version contains supplementary material available at 10.1007/s10238-024-01521-x.

## Background

Hepatocellular carcinoma (HCC) is a common solid primary liver tumor. It is ranked the sixth among cancers worldwide [1, 2], with a five-year survival rate of nearly 18% [3]. Many risk factors contribute to HCC, such as viral hepatitis, liver cirrhosis, alcohol consumption, non-alcoholic steatohepatitis, and aflatoxins [4]. Hypoxia is a critical factor in the tumor microenvironment (TME) of solid tumors because of the high proliferation rate of the tumor cells and impairment of the oxygen supply, and distant blood vasculatures or their temporary collapse [5]. This shortage of blood supply causes oxygen levels in the hypoxic tumor cells to be as low as 1–2% [6, 7]. Hypoxia profoundly affects various cancer-related properties such as angiogenesis, proliferation, genomic instability, immune evasion, resistance to therapy, metabolic switch, stemness, invasion, and metastasis [8–13]. The impact of hypoxia in the TME is achieved by hypoxia-inducible factors (HIFs). HIFs are heterodimeric transcription factors comprising of two subunits, *α* and *β* subunits, which are the inducible and constitutively expressed subunits, respectively [14]. The *α* subunit exists in three isoforms, which are HIF-1A, HIF-2A, and HIF-3A. The instability of HIF-1A and HIF-2A during normoxia is due to prolyl hydroxylation by prolyl hydroxylase domain-containing proteins (PHDs) that will be subsequently recognized by von Hippel-Lindau tumor suppressor protein (VHL), leading to ubiquitination of those subunits and then proteasomal degradation [15]. Contrarily, during hypoxia, the whole degradation process is hindered, so HIF-1A and HIF-2A translocate into the nucleus, heterodimerize with HIF-B; then, the whole HIFs bind to the hypoxia-responsive element (HRE) at the promoter of their downstream targeted genes [16, 17].

Many clinical studies reported a strong association between HIF-1A and poor prognosis in HCC patients [18–20]. It was also demonstrated that HIF-2A and HIF-3A expressions are less relevant to poor clinical outcomes [21–23]. Those studies denoted a significant correlation between the expression of HIF-1A and HCC hallmarks, including metabolism, angiogenesis, metastasis, immune evasion, and cancer stem cells. Under normal physiological conditions, the cells utilize glucose to produce pyruvate, which enters the mitochondria for further metabolism via tricarboxylic acid cycle (TCA) and oxidative phosphorylation (OXPHOS) to produce ATP. However, during hypoxia, the produced pyruvate is subsequently metabolized to lactate through anaerobic glycolysis [24]. This is dependent on HIF-1A as one of the critical key accelerators of glycolysis through its action on the downstream targeted genes [25, 26].

In HCC, overexpression of HIF-1A increases the activity of glycolytic enzymes such as enolase 1 (ENO1), lactate dehydrogenase A chain (LDHA), 6-phosphofructo-2-kinase/fructose-2,6-bisphosphate 3 (PFKFB3), hexokinases (HK1 and HK2), glyceraldehyde 3-phosphate dehydrogenase (GAPDH), phosphofructokinase-liver type (PFKL), and aldolases (ALD-A and ALD-C) [27]. In addition to increasing glucose metabolism, HIF-1A enhances glucose uptake by the tumor cells by inducing the activity of glucose transporter proteins GLUT1 and GLUT3 [28]. In the same way, HIF-1A upregulates the expression of pyruvate dehydrogenase kinase 1 (PDK1), which inhibits pyruvate conversion into acetyl coenzyme A (acetyl-CoA), resulting in the inhibition of TCA and OXPHOS in the mitochondria [29].

Computational approaches to identify the HIF-1A targeted genes and activated pathways were undertaken to study the genetic signature of hypoxia-related genes in HCC [30]. Bai et al. used univariate Cox regression analysis followed by a random forest feature selection algorithm to screen the prognostic genes in hypoxic conditions. The authors developed a prognostic model that predicts the overall patient’s survival based on multivariate Cox regression analysis of selected genes. Moreover, they employed Gene Set Enrichment Analysis (GSEA) to investigate enriched pathways in the development and progression of HCC. They validated using their prognostic model as a potential diagnostic indicator for HCC patients through Kaplan–Meier survival analysis, facilitating personalized medical decisions. The Cox regression analyses and the risk model approaches were also employed by Tang et al*.* [31] to identify hypoxia-related prognostic genes in HCC after conducting differential expression analysis to determine the molecular mechanisms of hypoxia-related genes in HCC. They obtained prognostic signatures, including eight hypoxia-associated genes (ENO2, KDELR3, PFKP, SLC2A1, PGF, PPFIA4, SAP30, and TKTL1). The different levels of HIF-1A expression have been used to represent hypoxia in cancer cells [32] and demonstrate hypoxia-mediated alterations in the expression of genes involved in the glycolysis pathway, specifically in lactate accumulation. The group investigated the quantitative effect of HIF-1A-deviated expression on specific glycolysis enzymes using mathematical modeling of a set of ordinary differential equations of their expressions. Their findings proposed phosphofructokinase-1 (PFK-1) and phosphoglucomutase (PGCM) as two enzymes that significantly contribute to the regulation of lactate production.

Experimentally, liver cancer organoids emerged as a relevant and personalized disease model to recapitulate the TME. In 2019, 129 FDA-approved chemotherapeutic drugs were tested using HCC organoids from patients’ specimens. Interestingly, only 9 of those drugs were reported to be effective across all lines of organoids [33]. In 2022, another study generated 52 HCC organoids derived from primary liver tumors of 153 patients. The results demonstrated that HCC organoids were better than patient-derived xenografts in experimental animals for drug screening. The study showed that sorafenib-acquired resistance in HCC organoids leads to epithelial–mesenchymal transition, retro-differentiation, and stemness, contributing to tumor aggressiveness [34].

Traditional 2D cell cultures have failed to mimic the in vivo TME to investigate hypoxia, particularly in generating high variability of oxygen tension in the tumor and its surrounding area [35]. Organoids, on the other hand, are 3D in vitro models that are potentially powerful tools to evaluate the effect of hypoxia in the TME [36]. Multicellular organoids encompassing tumor cells and surrounding stromal cells have been proposed to better represent the tumor hypoxic microenvironment. In a study by Wang et al*.* HCC organoids, including non-parenchymal cells such as fibroblasts and endothelial cells, displayed higher expression of HIF-1A, vascular endothelial growth factor (VEGF), transforming growth factor-*β* (TGF-*β*), vimentin, matrix metallopeptidase 9 (MMP9), and tumor necrotic factor-*α* (TNF-*α*) compared to organoids generated from HCC cells alone [37].

The two most employed methods to induce hypoxia are either via using a hypoxic chamber or chemical inducers such as CoCl_2_. The mechanism behind using CoCl_2_ as a chemical inducer for hypoxia is still under investigation; however, it was proposed that the Co^+2^ ion either replaces ferrous (II) iron or inhibits the reduction of ferric (III) iron into ferrous (II) iron in the active site of PHDs proteins or binds directly to HIFs and inhibits their further ubiquitination or degradation. The action of CoCl_2_ in mimicking the hypoxic conditions is thus achieved by stabilizing the HIFs [38].

Although using CoCl_2_ to induce hypoxic conditions requires further optimization for the duration of exposure and concentration to ensure the establishment of hypoxic conditions without compromising cell viability, it has many advantages such as affordability, availability, and consistency in maintaining the hypoxic-like conditions [39]. More importantly, we employed the chemical induction method using CoCl_2_ as it stabilizes explicitly the expression of the HIF-1A subunit without activating HIF-2A in liver cancer cell lines [40].

Although HIFs are considered the primary and robust regulators of hypoxia that modulate the effect of hypoxia by targeting the HREs in the downstream genes, there are also HIF-independent mechanisms that mediate the hypoxic effect; for example, Adamski et al*.* reported that in the hypoxic microenvironment of osteosarcoma, the level of phosphorylated p53 (an active form of p53) decreased when compared to normoxic conditions which results in the reduction of the chemotherapy-induced apoptosis, and this action is HIF-1A-independent as it is neither stimulated nor reversed by expression or inhibition of HIF-1A [41]. Additionally, in an HIF-1-independent manner, hypoxia induces the anti-apoptotic protein Pim1 of the pancreatic cancer cells, which in return suppresses the activity of two proteases important for cell death, which are caspase-9 and caspase-3 [42]. All the mentioned mechanisms mainly contribute to the chemotherapeutic resistance of cancer cells under hypoxic conditions. Some recent studies also demonstrated that hypoxia-induced non-coding RNAs might play an essential role in hypoxia-mediated apoptosis in an HIF-independent manner, such as circELP3 (hsa_circ_0001785) and circUBE2D2 (hsa_circ_0005728) in bladder cancer and HCC, respectively [43, 44].

Interestingly, the expression of CD133 as a cancer stem cell maker is also known to be upregulated both dependently and independently on HIF signaling; although CD133-positive cells were found in the tumor hypoxic regions of colon and rectal cancers, CD133 expression is inversely correlated to HIF-1A expression in patients who received preoperative chemotherapy. Accordingly, further investigations will be needed to unveil the molecular mechanisms of HIF-independent effects in the hypoxic TME of cancers [45].

In this manuscript, we are developing an in vitro HCC organoid model that recapitulates the role of HIF-1A specifically in HCC. HIF-1A is expressed in many solid tumors and is considered the main transcription factor that renders tumors the adaptation under hypoxia because it regulates the expression of more than 100 downstream genes involved in angiogenesis, metabolism, proliferation, and metastasis [46]. Moreover, in a study by Yang et al*.*, HIF-1A was significantly expressed in nearly 57% of tumor samples compared to only 5.6% in peritumoral tissues; more importantly, the expression of HIF-1A, not HIF-2A, is positively correlated to cancer cells infiltration and invasion, and metastasis [47].

**Fig. 1:**
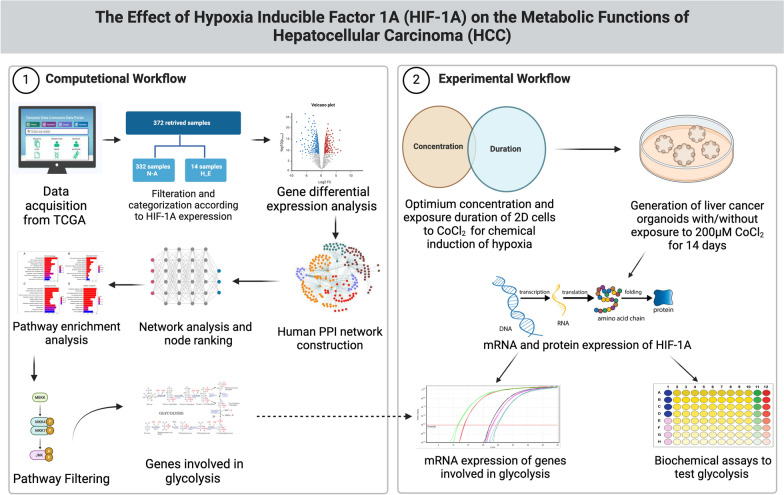
A schematic representation of the proposed workflow. The computational workflow starts with data acquisition from TCGA, where individuals are divided into groups which are highly expressed (H_E) and not altered (N-A) based on HIF-1A expression alterations. Differential expression analysis is then performed to detect genes whose expressions are altered comparably to HIF-1A. Next, a protein–protein interaction (PPI) network is utilized to rank the identified differentially expressed genes (DEGs). Pathway enrichment analysis is finally performed to highlight and correlate DEGs according to their known biological functions. The enriched pathways are then filtered based on their biological functions and their correlation to HIF-1A. The experimental section subsequently utilizes the DEGs involved in those filtered pathways. In this part, the optimum concentration of CoCl_2_ and duration of exposure of 2D cells to CoCl_2_ were identified to induce hypoxia chemically. Afterward, liver cancer organoids were generated with/without exposure to 200 µM CoCl_2_ for 14 days (optimum conditions), followed by further assessment of the mRNA and protein expression levels of HIF-1A to confirm the establishment of 3D in vitro model that displays hypoxic conditions. Finally, the results obtained from the computational work were validated by measuring the mRNA expression levels for the genes involved in glycolysis and performing biochemical assays to test the glycolytic functions of the generated organoids. The figure was created using BioRender (https://www.biorender.com/)

Accordingly, this study aims to develop a 3D in vitro organoid model to mimic the hypoxic conditions in the TME and to study the role of HIF-1A metabolic role in HCC without employing chemical inducers for hypoxia such as CoCl_2_ that might affect the development and growth of organoids. The validity of the constructed HCC organoid model grown spontaneously without CoCl_2_ was confirmed by the upregulated expression of HIF-1A and its downstream glycolytic genes obtained from the RNA-seq enriched pathways, which resulted in higher glucose consumption, intracellular pyruvate, lactate dehydrogenase expression, and extracellular lactate production. The developed HCC organoid model grown spontaneously represents a reliable model to study the hypoxic microenvironment and can be employed on a large scale for high-throughput screening for therapeutics targeting HIF-1A and its downstream genes, which are considered as potential prognostic markers in HCC patients (Fig. 1).

## Methods

### Data acquisition and filtration

Liver hepatocellular carcinoma (LIHC) RNA-Seq data used in this study were obtained from the Cancer Genome Atlas (TCGA) (project ID: TCGA-LIHC^1^). Only primary tumor samples were employed to eliminate any bias or distortion in the data. The total number of primary tumor samples was 377. Data Commons Bioconductor package^2^ was used to download the raw transcriptome profiling data. cBio Cancer Genomics Portal^3^ [48, 49], an open resource for analyzing cancer genomics datasets, was used to categorize samples according to the HIF-1A expression. Only 372 samples were available for retrieval. A z-score threshold of ± 2.0 was set to calculate the standard deviations from the mean expression of HIF-1A in all samples as a reference population**.** The samples were categorized into five subgroups, where 14 samples have high HIF-1A expression, eight samples have low HIF-1A expression, two samples have mutated HIF-1A gene, 16 samples are not profiled either in mutation, putative copy, fusion, or mRNA expression, and 332 samples have unaltered expression as illustrated in Online Resource 1. To study the effect of HIF-1A on the cancer-related properties in HCC patients, only the Highly Expressed (H_E) versus the Not Altered (N_A) groups were included in further data (Online Resource 2). Low HIF-1A expression cases were eliminated since they display genetic profiles different from those of the not altered cases [50, 51]. The final number of utilized samples was 346 after filtration. Principal component analysis (PCA) plots have been utilized, using plotPCA from DESeq2^4^ package (V ‘1.32.0’) [52], to evaluate the impact of differences in demographic factors such as age group, gender, ethnicity, and race between H_E and N_A on the gene expression profile as a source of variation. The age of 60 was used to split patients into two age groups. The results were visualized using “ggplot2” (V ‘3.5.1’)^5^ R package [53].

### Differential gene expression analysis

Differential expression analysis was conducted via DESeq2 (V ‘1.32.0’) comparing samples of H_E and N_A. DESeq2 employs the median of the ratios technique to normalize raw gene counts [52]. DESeq2 outperformed 11 RNA-seq differential analysis methods in terms of false discovery rate (FDR) control, power, and stability across all sample sizes in the benchmarking study conducted by Dongmei Li et al. A negative binomial generalized linear model was applied to estimate the dispersion of gene read counts [54]. Differential analysis was computed through a two-tailed Wald test. Statistically significant DEGs were identified using adjusted *P* values (*P*_adj_) and Log2 Fold Change (Log2FC), with a significance threshold set at Padj < 0.01 and an absolute Log2FC value greater than 2.

### Protein–protein interaction (PPI) network construction and visualization

As a baseline for constructing PPI networks, investigating the interactions between proteins indicates essential insights related to protein function or their organizational rules [55]. The PPI network was constructed using the identified DEGs through the STRING database (V 11.5)^6^[56]. STRING is an online search tool that retrieves the interactions between genes/proteins [56]. An interaction confidence score of 0.7 or above was set. The network was exported to Cytoscape (V 3.8.0) 6 [57] for network visualization and analysis. Cytoscape plugin Network Analyzer^7^ [58] was used to compute the topological parameters, such as nodes’ degrees. The degree of a node represents the number of interactions the protein has with other proteins, reflecting its importance. The nodes were sorted in descending order using their degree for pathway enrichment analysis.

### Pathway enrichment analysis

Pathway enrichment analysis was performed utilizing G: Profiler version *e108_eg55_p17_d098162*^8^ [59], where biological pathways with Benjamini–Hochberg FDR < 0.01 were considered significantly enriched. Only the enriched pathways with HIF-1A among their genes were retrieved, and the pathways involved in the metabolic functions were subjected to further analysis.

### Kaplan–Meier survival analysis

The Kaplan–Meier method with a log-rank statistical test was used to conduct survival analysis and identify genes with potential prognostic value. HIF-1A highly expressed (H_E) and non-altered (N_A) groups were used to investigate the association between HIF-1A expression and overall patients’ survival. Other targeted genes were also tested to assess their prognostic potential. The median of their expression levels was employed to categorize the samples into high- or low-expression groups. Genes with a *p* value threshold of < 0.05 were considered to have significant prognostic potential. All analyses were performed using the “survival” R package (V ‘3.5.7’)^9^ [60], “ggplot2” (V ‘3.5.1’) [53], “survminer” (V ‘0.4.9’)^10^ [61], and “cowplot” (V ‘1.1.3’)^11^ [62] R packages to calculate the survival curves, perform statistical tests, and visualize the results.

### Cell lines and cell culture

Huh7 cell line and human bone marrow-derived mesenchymal stem cells (hBM-MSCs) (ATCC, USA) were cultured in Dulbecco’s modified Eagle medium (DMEM) (Serana, Germany) with supplements of 10% fetal bovine serum (FBS) (Biowest, France), 1% penicillin-streptomycin-amphotericin B (Serana, Germany), and 0.5% L-glutamine (Corning, USA), while human umbilical vein endothelial cells (HUVEC) (Thermo Fisher Scientific, USA) were cultured in DMEM/F12 (Biowest, France) with supplements of 20% FBS, 2% L-glutamine, 2% penicillin-streptomycin-amphotericin B, 0.1% of 1000 IU/ml heparin (Nile, Egypt), 0.0072% of 1000 IU/ml insulin (Acros Organics, USA), 0.005% of each of 10 µg/ml basic fibroblast growth factor (b-FGF) and epidermal growth factor (EGF) (Pepro Tech, UK), 0.0025% of 1 mg/ml ascorbic acid (Sigma-Aldrich, USA), and 0.002% of 1 mg/5 ml dexamethasone (Serva, Germany). For the induction for hypoxic conditions, 25 mM CoCl_2_ stock (Loba Chemie, India) was prepared followed by preparing cell culture media with 50, 100, and 200 µM of CoCl_2_ to be added for 24 h to the cultured cells [63]. The cells were kept at 5% CO_2_ and 37 °C, and the cultured media were changed every 3 days.

### Matrix preparation and organoid generation

To generate the HCC organoids inside ECM, a modification of Sadeghi-Ataabadi’s protocol was applied [64] as 5 × 10^3^ cells of Huh7, HUVEC, and BM-MSCs were added and suspended in 50 µl of 50% platelets-rich plasma (PRP), 45% DMEM high glucose, and 5% of 3% calcium (II) chloride (CaCl_2_) (Alpha Chemika, India), then were cultured in a dome-like shape in 24-well plate and kept at 37 °C till complete solidification of ECM. Afterward, the ECM including the cells was cultured in Huh7 media and HUVEC media with ratio 1:1 [65]. For the induction for hypoxia-like conditions, 25 mM CoCl_2_ stock (Loba Chemie, India) was prepared followed by preparing organoid culture media with 200 µM of CoCl_2_. The cells were kept at 37 °C and 5% CO_2_ incubator for 14 days, and the cultured media with and without the 200 µM of CoCl_2_ were changed every three days. The length of the generated organoids was measured using ImageJ 1.53 K software.

### HCC organoid collection

The generated HCC organoids were washed with 1 × PBS (Loba Chemie, India) for 10–15 min and then collected via incubation with 1.5% Trypsin (Serana, Germany) at 37 °C till complete dissolving of the ECM. Then, the trypsin action was inhibited using complete culture medium (CCM) and the cell suspension was centrifuged for 10 min at 2000 RPM at 15 °C. Then the cell pellet was washed twice with 1 × phosphate buffer saline (PBS) and centrifugation for another 10 min for each wash.

### MTT assay

The MTT solution was prepared as 5 mg of MTT powder (SERVA, Germany) was dissolved in 1 ml 1 × PBS. The MTT solution was added to the 2D cells or to the HCC organoids after their retrieval from the scaffold, followed by 3-h incubation at 37 °C and 5% CO_2_. The formed formazan salts were dissolved using Dimethyl Sulfoxide (DMSO) (Serva, Germany) for 15 min of continuous shaking [66]. The optical density (OD) was measured at 490 nm with FLUOstar Omega microplate reader (BMG Labtech, Germany).

### Cell viability assay

After organoid collection, the pellet was resuspended in a 200 µl FACS buffer with 10 µl of 50 µg/ml propidium iodide (PI) [67]. The suspension was transferred into falcon tubes (Becton Dickinson, USA) to be incubated in dark for 15 min at room temperature. Cell viability was assessed using flow cytometer (Becton Dickinson, USA), and data were analyzed by FlowJo v.10.8.0 software.

### Real-time qPCR

According to the manufacturer’s protocols, mRNA extraction from 2D cells and organoids was performed using TRIzol reagent (Thermo Fisher Scientific, USA), followed by synthesis of cDNA with Revert Aid First Strand cDNA Synthesis Kit (Thermo Fisher Scientific, USA). The RT-PCR was done using HERA PLUS qPCR SYPER Green kit (Willowfort, UK). The 2^−ΔΔct^ method was applied to calculate the relative gene expression, and HPRT1 gene was employed as a housekeeping gene for normalization [68]. Each reaction was repeated three times, and each experiment was performed twice. Table 1 shows the sequences of the used primers.

**Table 1:** Sequence of the primers used

Name	F/R	Sequence	Name	F/R	Sequence
HPRT1	F	5’-CGGCGACGACCCATTCGAAC-3’	ENO2	F	5’-CTGTATCGCCACATTGCTCAGC-3’
	R	5’-GAATCGAACCCTGATTCCCCGTC-3’		R	5’-AGCTTGTTGCCAGCATGAGAGC-3’
HIF-1A	F	5’-CATAAAGTCTGCAACATGGAAGGT-3’	ENO3	F	5’-TGGGAAGGATGCCACCAATGTG-3’
	R	5’-ATTTGATGGGTGAGGAATGGGTT-3’		R	5’-GCGATAGAACTCAGATGCTGCC-3’
HK2	F	5’-GAGTTTGACCTGGATGTGGTTGC-3’	PFKFB3	F	5’-CAGTTGTGGCCTCCAATATC-3’
	R	5’-CCTCCATGTAGCAGGCATTGCT-3’		R	5’-GGCTTCATAGCAACTGATCC-3’
PKM	F	5’-ATGGCTGACACATTCCTGGAGC-3’	PFKP	F	5’-AGGCAGTCATCGCCTTGCTAGA-3’
	R	5’-CCTTCAACGTCTCCACTGATCG-3’		R	5’-ATCGCCTTCTGCACATCCTGAG-3’
GCK	F	5’-CATCTCCGACTTCCTGGACAAG-3’	SLC2A2 (GLUT2)	F	5’-ATGTCAGTGGGACTTGTGCTGC-3’
	R	5’-TGGTCCAGTTGAGAAGGATGCC-3’		R	5’-AACTCAGCCACCATGAACCAGG-3’
SLC2A1 (GLUT1)	F	5’-TTGCAGGCTTCTCCAACTGGAC-3’	LDHA	F	5’-GGATCTCCAACATGGCAGCCTT-3’
	R	5’-CAGAACCAGGAGCACAGTGAAG-3’		R	5’-AGACGGCTTTCTCCCTCTTGCT-3’
P53	F	5′-GTTCCGAGAGCTGAATGAGG-3′	P21	F	5′-TGGAACTTCGACTTTGTCAC-3’
	R	5′-TTATGGCGGGAGGTAGACTG-3′		R	5′-CACATGGTCTTCCTCTGCT-3’
YWHAB	F	5′-CTGAAGTGGCATCTGGAGACAAC-3′			
	R	5′-GACGAATTGGGTGTGTAGGCTG-3′			

### Immunofluorescence staining

The HCC organoids were washed 3 times with 1 × PBS, each 10 min. For permeabilization, 0.3% Triton x-100 (Sigma-Aldrich, USA) was added at room temperature for 15 min. Then, the organoids were washed for 3 times with 1 × PBS, each was 10 min. For blocking, 5% bovine serum albumin (BSA) (Sigma-Aldrich, USA) was added for 2 h at room temperature. Anti-human HIF-1A (Cell Signaling, USA) was diluted in blocking solution to the recommended concentration according to the manufacturer’s instructions and incubated with organoids for 16 h at 2–8 °C in the dark. The organoids were then washed with 1 × PBS, 3 times/10 min, and Alexa Fluor 555 goat anti-mouse IgG (Life Technologies, USA) was diluted in blocking solution to the recommended concentration according to the manufacturer’s instructions and incubated with the organoids in a dark humidified place at room temperature for 2 h. The organoids were washed 3 times with 1 × PBS, each for 10 min; then, 3 µg/ml Hoechst 33,342 (Life Technologies, USA) as a counterstain was added for 15 min at room temperature in a dark, humidified place. The organoids were then washed for 3 times with 1 × PBS, each was 10 min. Visualization was performed using Leica inverted fluorescent microscope (Leica Microsystems, Germany); data analysis was done using ImageJ 1.53 K software.

### Glucose consumption

The HCC organoid culture media in both organoid groups, with and without 200 µM CoCl_2_, were collected from day 11 to day 14 of organoid generation. According to the manufacturer’s protocol (Biodiagnostic, Egypt), 200 µl of working solution was added to 2 µl of standard and organoid culture media containing 24-well plate and incubated at 37 °C for 10 min. The working solution was prepared by mixing phenol as chromogen, phosphate buffer, 4-amino antipyrine, glucose oxidase, and peroxidase. The absorbance of the sample and standard was measured against the blank at 510 nm using FLUOstar Omega microplate reader.

### Intracellular and extracellular pyruvate concentration

After HCC organoid retrieval of 3 scaffolds containing organoids per group, 100 µl of lysis buffer was added to the pellet, then vortexed for 10 min on ice, followed by centrifugation at 16,000 ×g at 4 °C for 20 min. For intracellular pyruvate concentration (Biochemical Enterprise, Italy), in a 24-well plate, 30 µl of cell lysate or standard was added to each well containing 171.5 µl working solution and incubated at 37 °C for 5 min; then, the first absorbance reading was measured at 340 nm; working solution was prepared by dissolving NADH in 20 ml of the buffer vial. Then, 26 µl of lactate dehydrogenase (LDH) was added and incubated at 37 °C for another 5 min. The second absorbance reading was measured at 340 nm. Both absorbance readings were measured against the blank using FLUOstar Omega microplate reader. For extracellular pyruvate concentration, the same protocol was applied using organoid culture media collected between day 11 and day 14 of the organoid generation period.

### Extracellular lactate concentration

The HCC organoid culture media in both organoid groups, with and without 200 µM CoCl_2_, were collected from day 11 to day 14 of the organoid generation period. According to the manufacturer’s protocol (Spectrum, Egypt), 100 µl of working reagent was added to 1 µl of standard and organoid culture media contained in 24-well plate and incubated at 37 °C for 5 min. The working reagent is composed of tris buffer, 2,4,6-tribromo-3-hydroxybenzoic acid, 4-amino antipyrine, lactate oxidase, peroxidase, and sodium azide. Both absorbance readings were measured against the blank at 546 nm using FLUOstar Omega microplate reader.

### NADPH assay

According to the manufacturer’s protocol (BioAssay Systems, USA), 100 µl NADPH extraction buffer was added to the pellet of HCC organoids after retrieval from ECM at 60 °C for 5 min; then, 100 µl opposite extraction buffer and 20 µl assay buffer were added to neutralize the extract. Then samples were subjected to brief vertexing and spinning at 14,000 rpm for 5 min. Then, 40 µl of the supernatant of each group was added per well and mixed briefly with 80 µl working reagent; the working reagent was prepared by mixing 60 µl assay buffer, 1 µl enzyme mix, 10 µl glucose, and 14 µl MTT for each well of the reaction. The OD was measured at zero time and after incubation at room temperature for 30 min at 565 nm using FLUOstar Omega microplate reader.

### ATP assay

According to the manufacturer’s protocol (Biovision, USA), 100 µl of ATP buffer and 500 µl of working solution (phenol: chloroform: deionized water at ratio 6:2:2, respectively) for deproteinization were added to the pellet of HCC organoids after retrieval from ECM. The mixture was shaken for 20 s and centrifuged at 10,000 ×g for 5 min at 4 °C; then, the upper aqueous layer was transferred to new Eppendorf. Then, 50 µl of the supernatant of each group was transferred to one well and 50 µl of reaction mix was added to each well with mixing and incubation at room temperature for 30 min. The reaction mix was made of 44 µl ATP assay buffer, 2 µl ATP probe, 2 µl ATP converter, and 2 µl developer for each well of the reaction. The OD was measured at 570-nm FLUOstar Omega microplate reader.

### Hydrogen peroxide assay

100 µl of HCC organoid culture media in both organoid groups, with and without 200 µM CoCl_2_, was added to 100 µl of chromogen (phosphate buffer pH 7.0 3,5 dichloro-2-hydroxy benzene sulfonate detergent), and 100 µl of enzyme (4–aminoantipyrine peroxidase preservative), followed by incubation at 37 °C for 10 min (Biodiagnostics, Egypt). Both absorbance readings of the sample and standard (dil. 1000 times before use) were read against the blank at 490 nm using FLUOstar Omega microplate reader.

### Statistical analysis

The data were presented as mean ± SD. Significance was calculated via a two-tailed *T* test using IBM SPSS Statistics version 23.0.0.0. *P* < 0.05 was considered statistically significant.

## Results

### Analysis of demographic variation effects

**Fig. 2:**
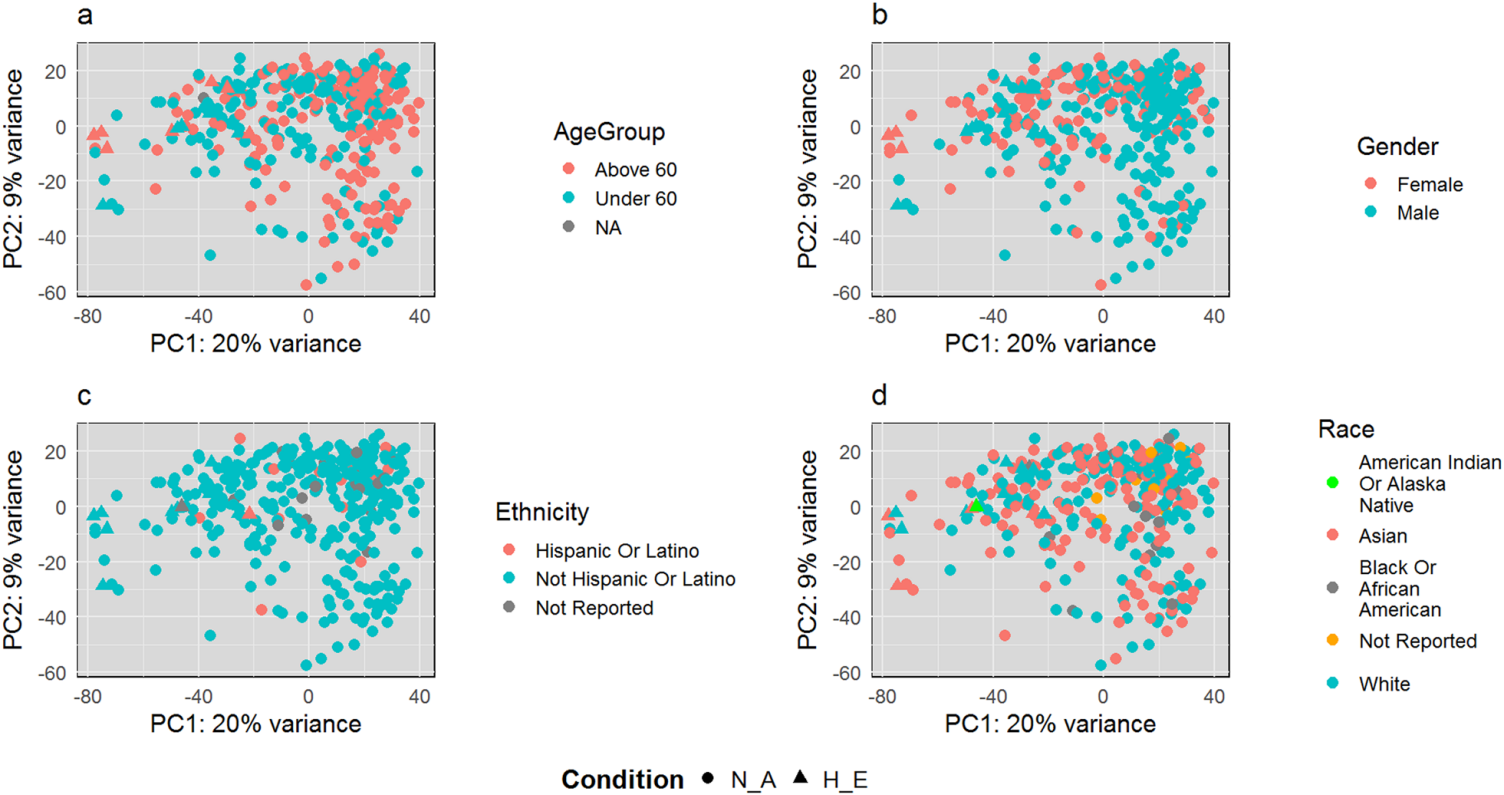
PCA plots for the demographic factors of H_E and N_A groups. **a** Age group, **b** gender, **c** ethnicity, and **d** race have no confounding effects on gene expression variations between the H_E and N_A groups

The total number of samples was 346, which includes 14 samples in the (H_E) group versus 332 in the (N_A) group. Demographic data include age, gender, ethnicity, and race. Table 2 summarizes all demographic information. The data have 232 males and 114 females. The age range was from 16 to 88 years. PCA illustrates that age, gender, ethnicity, and race are not considered confounding factors that cause gene expression variations between the H_E and N_A groups. As shown in Fig. 2, no trends, patterns, or clusters are shaped according to these demographic values' distributions.

**Table 2:** Demographic information

Gender		Race				Ethnicity			Age			
Male	Female	White	Asian	American Indian or Alaska Native	Black or African-American	Not Hispanic or Latino	Not Reported	Hispanic or Latino	Mean	SD	Minimum of range	Maximum of range
232	114	170	149	1	16	312	18	16	59 years and 242 days	13 years and 81 days	16 years and 22 days	88 years and 0 days

### Differential gene expression and PPI network construction

**Fig. 3:**
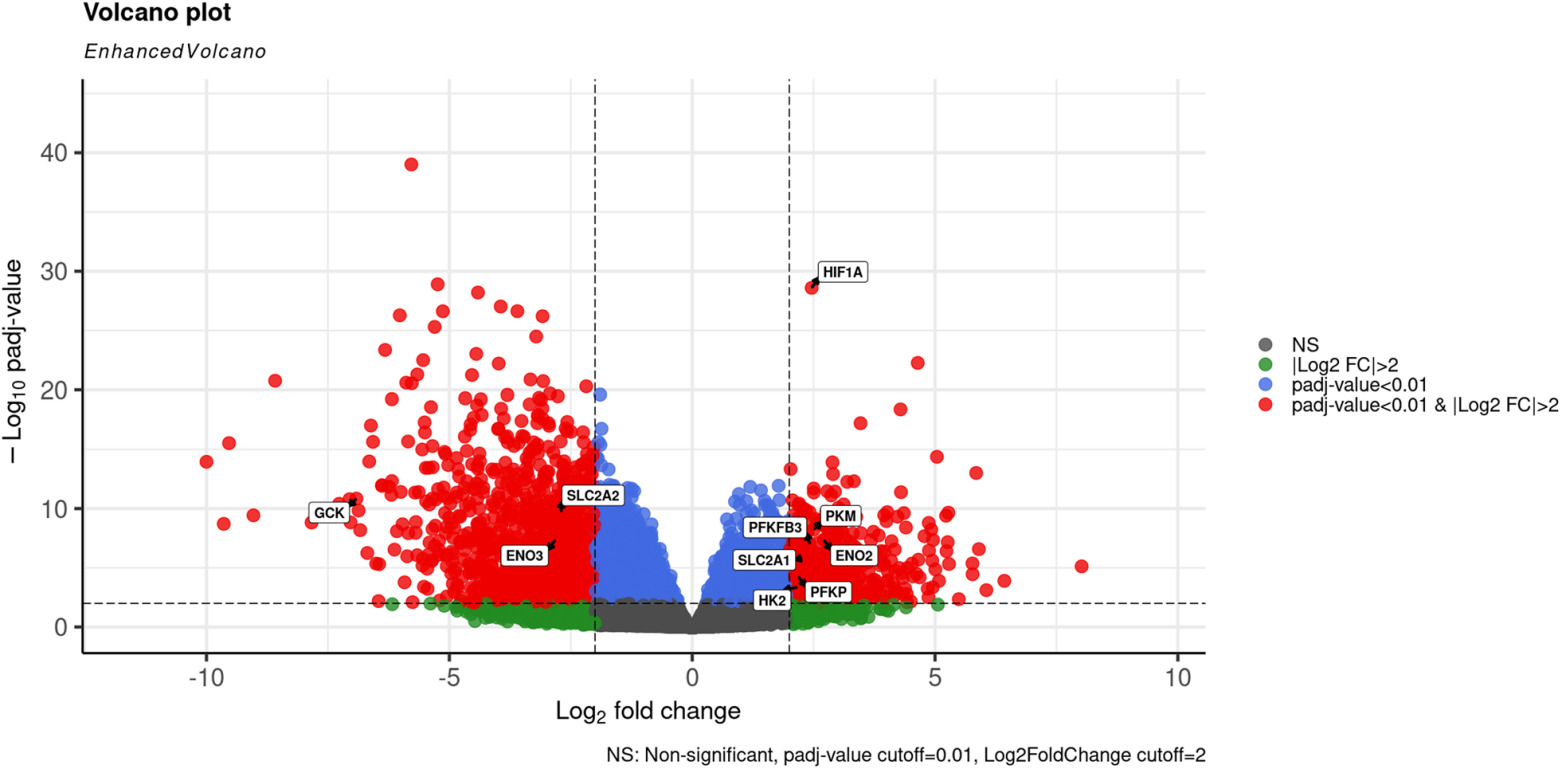
Volcano plot represents the differentially expressed genes. The differentially expressed genes with expression levels of a threshold |log2FC|> 2 and adjusted *P* value < 0.01. Red circles: DEGs with |Log2FC|> 2 and adjusted P-value < 0.01; blue circles: DEGs with adjusted *P* value < 0.01; green circles: DEGs with |Log2FC|> 2, gray circles: non-significantly expressed mRNAs. The HIF-1A and its target genes are labeled with a label box showing their gene expression level (Color figure online)

The TCGA-LIHC dataset was utilized to identify DEGs between the H_E and N_A groups. Utilizing 14 H_E and 332 N_A samples, 1852 DEGs were identified with a cutoff of |log2FC|> 2 and adjusted *P* value < 0.01 (Online Resource 3). Figure 3 shows the identified DEGs in red in the Volcano plot, where the positive log2 fold change reflects upregulated genes, and the negative log2 fold change reflects downregulated genes. As illustrated, more genes were downregulated (1313 genes) than upregulated (539 genes).

Utilizing STRING [56], a PPI network with 1136 proteins connected by 1713 edges was constructed. Nodes with zero degrees according to the predetermined significance threshold were excluded from the network diagram for better visualization (S_Figure 1). The final list of all genes with their degrees is illustrated in Online Resource 4.

### Pathway enrichment analysis

As HIF-1A regulates many genes related to diseases and carcinogenesis, the genes expressed in response to hypoxia contribute to only 5% of all human genes or less [69]. Pathway enrichment analysis has been performed on the identified genes of interest using the network degree ranking. A total of 327 enriched pathways were identified utilizing all database sources in G: Profiler (Online Resource 5) with False Discovery Rate (FDR) < 0.01. To study the hypoxic effects mediated by HIF-1A in HCC patients, only the pathways including HIF-1A among their genes were subjected to further investigation. Five pathways with 23 other genes were identified, as shown in Table 3. Pathways were categorized according to their corresponding biological function in the pathway source database. Two categories were identified: metabolism and signaling pathways, as illustrated in Table 3.

**Table 3:** Enriched pathways including HIF-1A

Pathway	Source	Biological function	Adjusted *p*-value	Genes
Central carbon metabolism in cancer	KEGG	Metabolism pathway	0.001134508523	PKM, HK2, HIF1A, SLC2A1, PFKP, GCK, SLC2A2, GLS2
HIF-1 signaling pathway	KEGG	Metabolism pathway	0.002726246027	IL6, HK2, HIF1A, ENO2, SLC2A1, PFKP, PFKFB3, CAMK2B, ENO3
Interleukin-4 and interleukin-13 signaling	REAC	Signaling pathway	0.003102074552	IL6, HK2, HIF1A, ENO2, SLC2A1, PFKP, PFKFB3, CAMK2B, ENO3
Clear cell renal cell carcinoma pathways	WP	Signaling pathway	0.0001689005564	PKM, HK2, PKLR, HIF1A, ENO2, SLC2A1, PFKP, ENO3, ENPP3, SDS
Vitamin D receptor pathway	WP	Signaling pathway	0.002841615312	CYP3A4, CYP1A1, CYP2C9, CYP2B6, CYP7A1, KNG1, HIF1A, CYP2D6

**Fig. 4:**
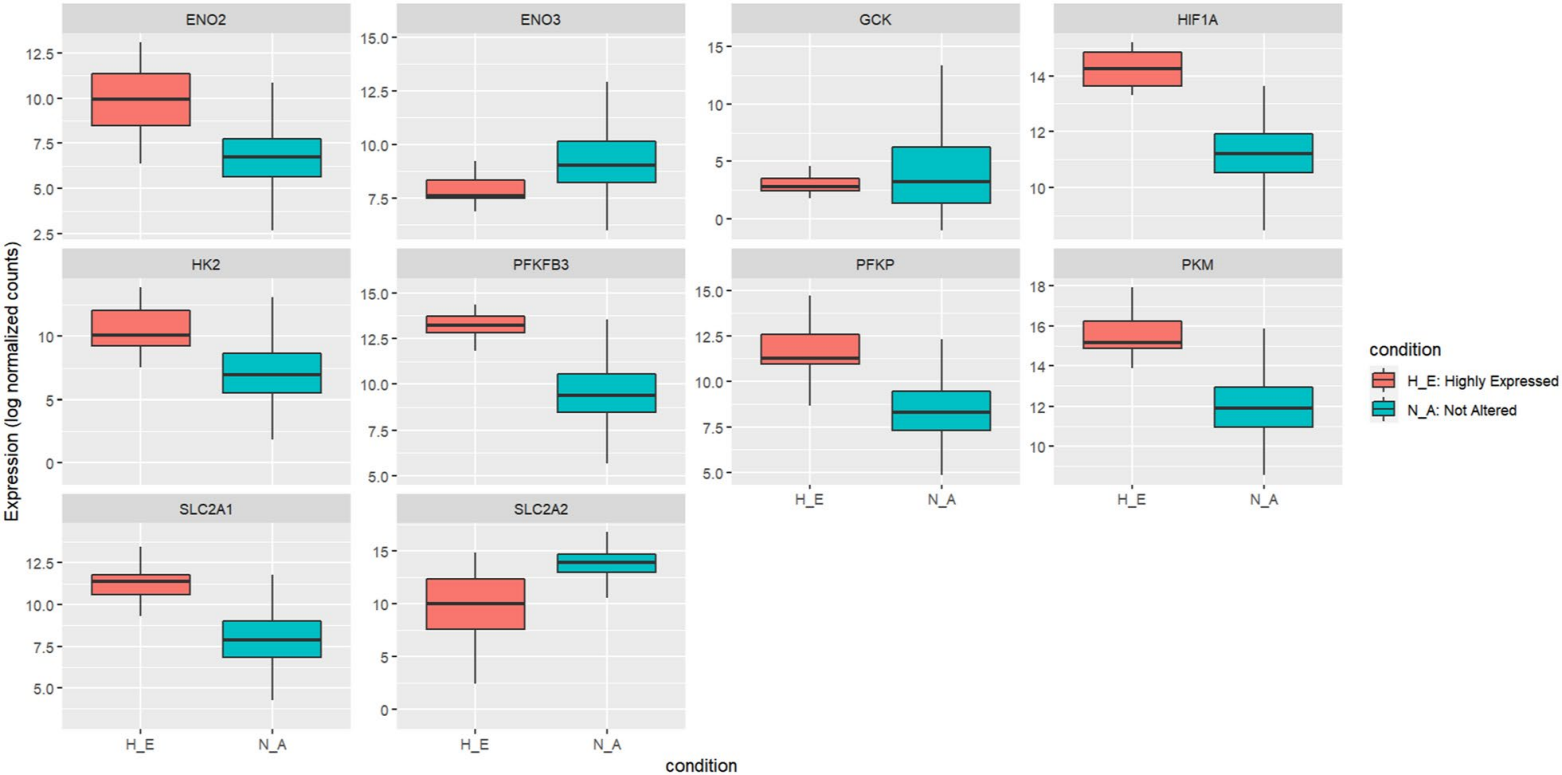
Boxplot diagrams of HIF-1A expression and its nine target genes. All boxplots have an X-axis representing the two conditions (N_A and H_E) and a Y-axis representing the gene expression for the samples toward each gene and their distribution among the two conditions. Not altered condition is represented in blue, while the highly expressed condition is represented in red (Color figure online)

**Fig. 5:**
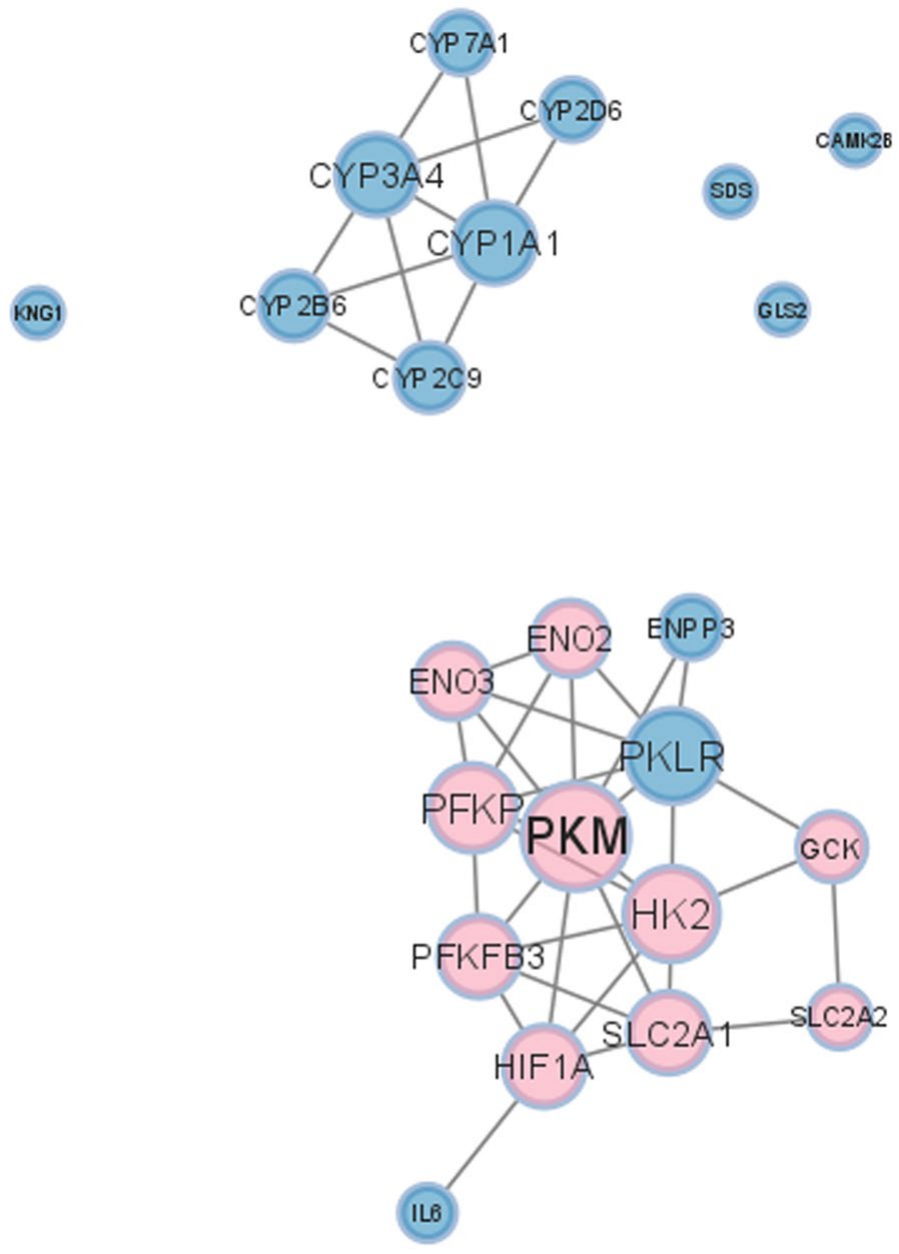
A sub-PPI network for genes in enriched pathways that include HIF-1A. The nine direct target genes in metabolism-related pathways are highlighted in pink including the HIF-1A. The other genes are colored in blue. The size of the node represents its degree in the original network (Color figure online)

More focus was given to central carbon metabolism in cancer and HIF-1 signaling pathways for their metabolic functions because glucose metabolism is one of the main cancer-related properties affected by HIF-1A [70, 71]. In hypoxic conditions, HIF-1A elevates, and its target genes are hence to be affected as well. In central carbon metabolism in cancer and HIF-1 signaling pathways, 9 out of 12 genes were identified as targets of HIF-1A, as shown in Table 4. The target genes were extracted through the pathway source database, where they were direct targets of HIF-1A. The expression of those target genes is more likely to be altered in cells under hypoxic conditions. Therefore, those genes were selected for further validation in a 3D in vitro liver cancer organoid model. Figure 4 illustrates the expression levels of the HIF-1A and its target genes in H_E samples versus N_A samples. HIF-1A and its target genes were also marked in the volcano plot in Fig. 3, representing the differentially expressed genes and their expression levels. Extracting the genes of interest from the original network in S_Figure 1, Fig. 5 shows a sub-network that includes the 23 genes separated into two clusters where our genes of interest are in the same cluster, which supports the direct relationship between them.

**Table 4:** Target genes of HIF-1A

Genes within metabolic pathways	HIF-1A targets genes
PKM	Central carbon metabolism in cancer
IL6	–
HK2	Central carbon metabolism in cancer/HIF-1 signaling pathway
SLC2A1	Central carbon metabolism in cancer/HIF-1 signaling pathway
ENO2	HIF-1 signaling pathway
GCK	Central carbon metabolism in cancer:
PFKFB3	HIF-1 signaling pathway
PFKP	Central carbon metabolism in cancer
SLC2A2	Central carbon metabolism in cancer
GLS2	–
CAMK2B	–
ENO3	HIF-1 signaling pathway

### HIF-1A and its targeted downstream genes as candidate prognostic markers for HCC

**Fig. 6:**
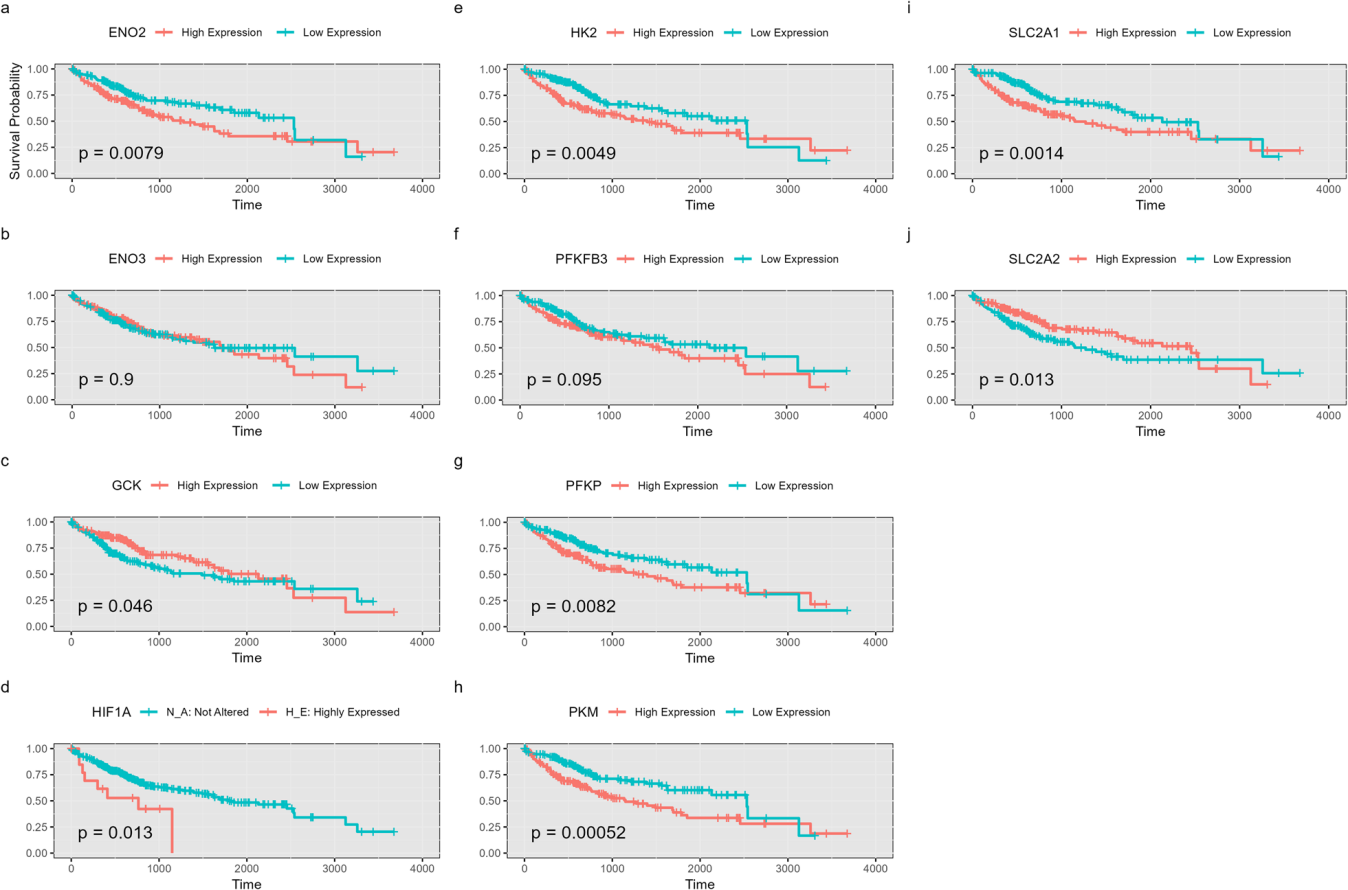
Kaplan–Meier survival curves for candidate genes. **a** ENO2, **b** ENO3, **c** GCK, **d** HIF-1A, **e** HK2, **f** PFKFB3, **g** PFKP, **h** PKM, **i** SLC2A1, **j** SLC2A2. Plots display the survival probability over time. The corresponding *p* values indicate statistical significance, and a *p* value threshold of < 0.05 is considered significant

Kaplan–Meier survival analysis results show a significant association between HIF-1A expression and patients’ survival outcomes. As illustrated in Fig. 6, HIF-1A high expression is correlated with a low survival rate (*p* value = 0.013), supporting using it as a potential prognostic marker in HCC. Figure 6 also illustrates a similar correlation between dysregulated gene expression and low survival rate for most candidate genes such as ENO2, GCK, HK2, PFKP, PKM, SLC2A1, and SLC2A2. Contrarily, no significant association is detected for ENO3 or PFKFB3 (Fig. 6b and f). Given that our study focuses mainly on HIF-1A as a candidate prognostic marker for HCC, we specifically chose pathways that include HIF-1A, particularly the pathways involved in metabolic reprogramming, as it is one of the main cancer-related properties affected by HIF-1A. Accordingly, we identified nine DEGs that we claim can be used as chemotherapeutic targets. Guided by this computational approach, our experimental work employs these nine DEGs to verify the reliability of our 3D HCC organoid model to simulate HIF-1A-mediated metabolic reprogramming.

### The optimum concentration of CoCl_2_ to induce the expression of HIF-1A

**Fig. 7:**
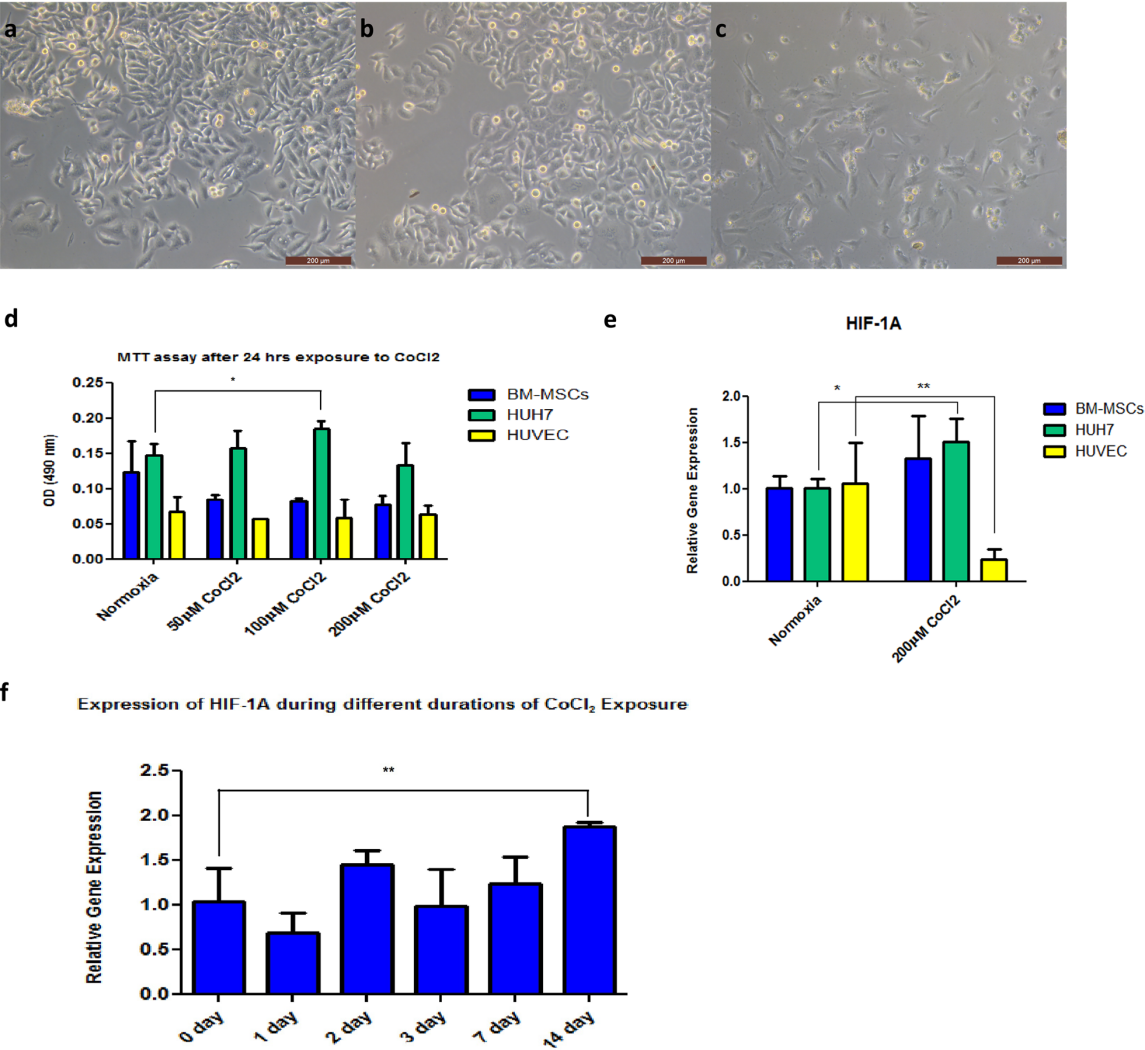
Effect of CoCl_2_ on proliferation and relative gene expression of HIF-1A in HCC cells. **a**, **b**, and **c** are bright-field micrographs of Huh7, BM-MSCs, and HUVEC, respectively (scale bar 200 µm). **d** is a bar graph of optical density (OD) at 490 nm from MTT assay of BM-MSCSs, Huh7, and HUVEC with 0, 50, 100, and 200 µM CoCl_2_. **e** is a bar graph from RT-PCR of the HIF-1A relative gene expression of BM-MSCSs, Huh7, and HUVEC with 0 and 200 µM CoCl_2_. f is a bar graph from RT-PCR for the relative gene expression of HIF-1A in Huh7 cells grown inside a plasma-derived scaffold for 14 days and exposed for 200 µM CoCl_2_ for 0, 1, 2, 3, 7, and 14 days. The represented values are mean ± SD, ^*^P < 0.05, ^**^P < 0.01

Huh7, BM-MSCs, and HUVEC were grown in 2D cell culture to evaluate their viability and HIF-1A gene expression during exposure to hypoxic-simulated conditions (Fig. 7a–c). MTT assay was employed to determine the concentration of CoCl_2_ that does not affect the cell proliferation of Huh7, BM-MSCs, and HUVEC (Fig. 7d). The data in Fig. 7d show a significant increase in the cell proliferation of Huh7 cultured in media supplemented with 100 µM CoCl_2_, while there is no significant difference in proliferation of the three cell types cultured with any of the CoCl_2_ concentrations. The relative gene expression of HIF-1A was measured in Huh7, BM-MSCs, and HUVEC grown in media supplemented with 0 and 200 µM CoCl_2_. Our data show a significant elevation in the HIF-1A gene expression level in Huh7 cultured in the 200 µM CoCl_2_ group, but a significant decrease in the gene expression of HIF-1A in HUVEC cultured under the same conditions (Fig. 7e). Huh7 cells grown as 2D cells but inside the plasma-derived scaffold for 14 days while exposed to 200 µM CoCl_2_ for 0 (control), 1, 2, 3, 7, and 14 days to identify the duration needed by the cells to express HIF-1A inside the ECM; accordingly, the data showed a significant elevation in HIF-1A expression only after 14 days of exposure to 200 µM CoCl_2_ compared with the control group (0 day) (Fig. 7f).

### The effect of CoCl_2_ on the size, viability, and proliferation ability of HCC organoids

**Fig. 8:**
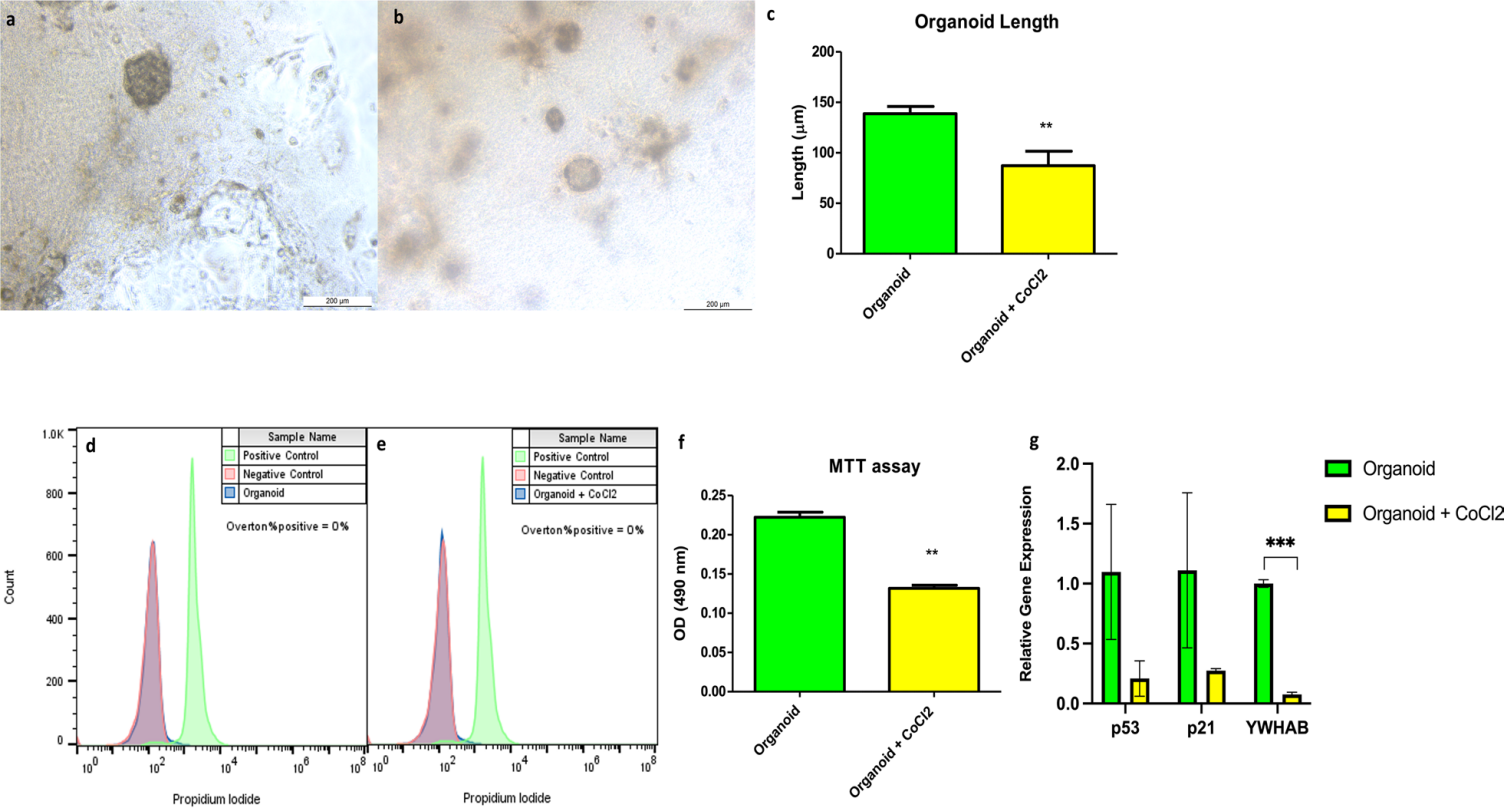
Generation of HCC organoids with and without CoCl_2_. **a** and **b** are bright-field micrographs for organoids grown in the absence or presence of 200 µM CoCl_2_ for 14 days, respectively (scale bar 200 µm). **c** is a bar graph for the length of the 2 organoid groups (the measured length is for 3 organoids/group, each measured 4 times). **d** and **e** are histograms for the viability of organoids grown in the absence or presence of 200 µM CoCl_2_ for 14 days, respectively (compared to positive and negative controls). **f** is a bar graph of OD at 490 nm from MTT assay for the 2 organoid groups. **g** is a bar graph from RT-PCR of the p53, p21, and YWHAB relative gene expression. The represented values are mean ± SD, *P < 0.05, **P < 0.01

The HCC organoids cultured in the absence of CoCl_2_ displayed a significantly larger diameter of nearly 138 µm than the ones cultured with 200 µM CoCl_2_, which had a diameter of nearly 86 µm (Fig. 8a–c). There was no significant difference in the viability of both groups of engineered organoids; the tested organoids in the absence and presence of 200 µM CoCl_2_ were compared with fixed dead and unstained cells as positive and negative controls, respectively (Figs. 8d and 6e). However, the proliferation rate/metabolic capacity of the organoids in the absence of CoCl_2_ was significantly higher than those in the presence of CoCl_2_ (Fig. 8f). Additionally, the relative gene expression analysis of p53 and p21 demonstrates no significant difference between the two HCC organoid groups, while HCC cultured in the absence of CoCl_2_ showed relatively higher gene expression of YWHAB than its counterpart (Fig. 8g).

### The effect of HIF-1A on the gene expression of its targeted genes involved in glycolytic pathways in HCC organoids

**Fig. 9:**
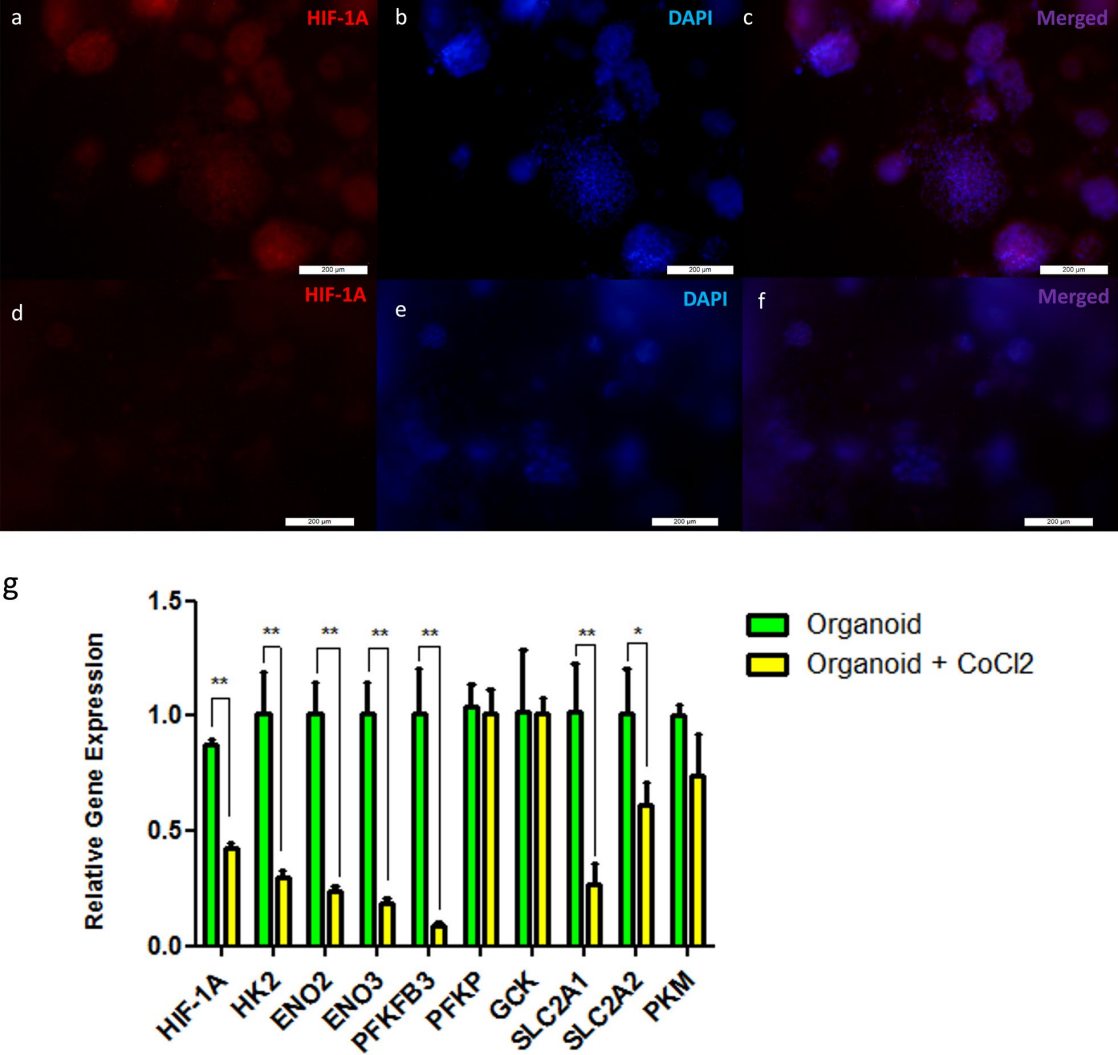
The expression levels of the HIF-1A and its targeted genes. **a**, **b**, and **c** are fluorescent micrographs for organoids, while **d**, **e**, and **f** are fluorescent micrographs for organoids with 200 µM CoCl_2_. **a** and **d** were stained with HIF-1A monoclonal antibody, and b and e were stained with Hoechst. **c** and **f** are merged fluorescent micrographs (scale bar 200 µm). **g** is bar graph from RT-PCR of the HIF-1A relative gene expression and its targeted genes such as HK2, ENO2, ENO3, PFKFB3, PFKP, GCK, SLC2A1, and SLC2A2, and PKM. The represented values are mean ± SD, ^*^P < 0.05, ^**^P < 0.01. *HK2* hexokinase 2, *PKM* pyruvate kinase M, *GCK* glucokinase, *ENO2* enolase 2, *ENO3* enolase 3, *PKKFB3 *6-phosphofructo-2-kinase/fructose-2,6-biphosphatase 3, *PFKP* phosphofructokinase, *SLC2A1* solute carrier family 2 member 1, and *SLC2A2* solute carrier family 2 member 2

Liver cancer organoids displayed higher expression of the HIF-1A gene and protein compared to those grown in the 200 µM CoCl_2_-containing media for 14 days (Fig. 9a–f and g). As shown in Fig. 4, the nine targeted genes of HIF-1A were subjected to further validation by determining their relative gene expression using RT-PCR (Fig. 9g). The bar graph illustrates a significantly higher gene expression of HK2, ENO2, ENO3, PFKFB3, SLC2A1, and SLC2A2 (Fig. 9g) in the HCC organoids that expressed HIF-1A compared to the organoids with lower HIF-1A expression. Furthermore, the data revealed no significant difference in the gene expression levels of PKM, GCK, and PFKP (Fig. 9g) between the two organoid groups.

### Elevation in the glycolytic activity of the HCC organoids under a hypoxic environment

**Fig. 10:**
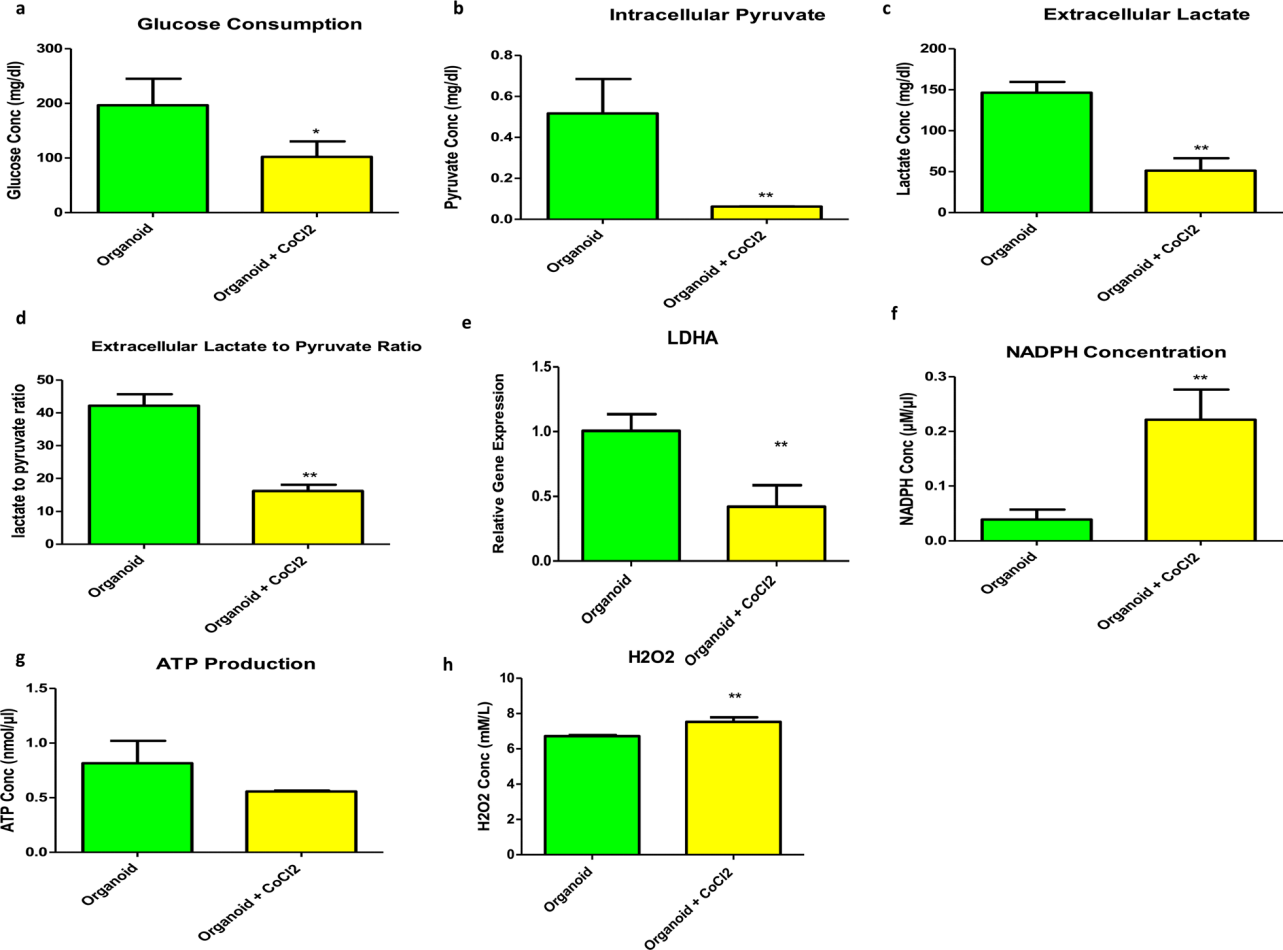
Glycolytic activity of HCC organoids in the presence or absence of hypoxic conditions. **a**, **c**, **f**, **g**, and **h** are bar graphs of the colorimetric assays to measure the concentration of glucose consumption, extracellular lactate, intracellular NADPH, ATP produced, and hydrogen peroxide, respectively. **b** is a bar graph of the spectrophotometric assay used to measure the intracellular pyruvate concentration. **d** is a bar graph of the colorimetric and spectrophotometric assays to measure the ratio of extracellular lactate to extracellular pyruvate. **e** is RT-PCR to determine the relative gene expression of LDHA. The represented values are mean ± SD, ^*^P < 0.05, ^**^P < 0.01

Liver cancer organoids with higher expression of HIF-1A showed more enhanced glycolytic activity than organoids cultured in 200 µM CoCl_2_ for 14 days (Fig. 10). The data of Fig. 10a–c and e revealed a significant increase in glucose consumption with a highly significant increase in the intracellular pyruvate and extracellular lactate production and gene expression level of LDHA in HCC organoids with higher expression levels of HIF-1A. Moreover, HCC organoids with higher HIF-1A expression demonstrated a significant elevation of extracellular lactate to extracellular pyruvate ratio compared to the other group with lower HIF-1A expression group (Fig. 10d). NADPH production was significantly lower under hypoxic conditions (Fig. 10f). There was no significant difference between the two groups regarding ATP production (Fig. 10g). Additionally, hydrogen peroxide concentration was significantly lower in the HCC organoid group with elevated HIF-1A expression (Fig. 10h).

## Discussion

Hypoxia in the TME contributes to the chemotherapeutic resistance of HCC to sorafenib (first-line treatment of liver cancer), doxorubicin, and cisplatin [72]; moreover, hypoxia plays an important role in tumor aggressiveness and poor prognosis [73]. Since HIF-1A is one of the key transcription factors that mediate the effect of hypoxia, it represents a promising target for chemotherapy. Our study aims to specifically identify HIF-1A targeted genes and activated pathways in HCC using computational approaches and to validate the obtained data using HCC organoids, which can be considered a reliable in vitro model to study the role of HIF-1A in the tumor microenvironment.

The expression of HIF-1A can be experimentally done using two approaches either a hypoxic chamber or chemical inducers such as cobalt (II) chloride hexahydrate (CoCl_2_.6H2O) [63]. In this study, we applied the chemical method using CoCl_2_ because of the similar expression patterns in the hypoxia-dependent genes, including glycolytic genes, between the two approaches [74]. More importantly, CoCl_2_ stabilizes HIF-1A and HIF-2A subunits. However, only the HIF-1A subunit is the functional subunit in HepG2 and Huh7 cell lines while using CoCl_2_[40]. Since the induction of HIF-1A expression using CoCl_2_ occurred in both time- and dose-dependent manner, we assessed its cytotoxicity using different concentrations on Huh7, BM-MSCs, and HUVEC after 24 h of exposure. 200 µM of CoCl_2_ was considered the most suitable concentration since it did not show a significant decrease or increase in the proliferation capacity of the three cell types (Fig. 7). It was also reported that higher doses reduce cell viability and proliferative capacity [75], while lower doses can lead to higher cellular induction [76].

200 µM of CoCl_2_ sufficiently induced the expression of the HIF-1A gene after 24 h in 2D Huh7 cell culture but not in BM-MSCs and HUVEC (Fig. 7). Previous studies reported a feedback mechanism induced by cell exposure to hypoxia, where some cells display a transient increase in HIF-1A expression for up to 6 h and then undergo downregulation in its expression during prolonged exposure to hypoxia for up to 36 h [77, 78]. Moreover, Jung et al*.* data presented that HUVEC show stable expression of HIF-1A just after 30 min of exposure to hypoxic conditions till maximum of 3 h which might explain the decline of HIF-1A expression in HUVEC after 24 h of exposure to CoCl_2_ [79]_._ Our data showed that the optimum dose of CoCl_2_-induced hypoxia in Huh7 cells cultured inside ECM was 200 µM, which optimally induced HIF-1A expression on day 14 of culture (Fig. 7).

In our study, HCC organoids cultured for 14 days in the absence of CoCl_2_ grew to a larger diameter of nearly 138 µm when compared with the other group of organoids cultured in 200 µM CoCl_2_ for the same period, which only attained a diameter of 86 µm. Similarly, the proliferation capacity and metabolic activity of HCC organoid groups without CoCl_2_ were significantly elevated. In contrast, both groups of organoids had no significant changes in their viability, suggesting that the applied concentration of CoCl_2_ affects the cell proliferation capacity without affecting organoid viability (Fig. 8). This may be due to the increase in the production of reactive oxygen species (ROS) such as hydrogen peroxide (Fig. 10h) with impairment in the DNA repair mechanisms caused by CoCl_2_, which subsequently led to DNA damage and cell death [80–82]. Also, the high proliferation rate in the absence of CoCl_2_ may be due to an increase in oxygen demand, leading to the hypoxic microenvironment of the organoid and stabilization of the expressed HIF-1A protein [83, 84]. In return, high levels of HIF-1A contribute to the uncontrolled proliferation and growth of HCC organoids, leading to a vicious cycle of hypoxia and cell proliferation [85–87]. Accordingly, the uncontrolled growth of HCC organoids in the absence of CoCl_2_ leads to the formation of a hypoxic microenvironment in those organoids compared to both 2D Huh7 cells and HCC organoids grown in the presence of CoCl_2_ in which the hypoxic microenvironment in 3D cultured cells such as spheroids and organoids is a well-known and widely reported phenomenon [88, 89]. Therefore, HCC organoids showed significantly higher expression of HIF-1A on both gene and protein levels compared to those cultured in CoCl_2_ (Fig. 9).

In our study, we validated the data obtained from the computational analysis of the TCGA-LIHC dataset. The retrieved data were categorized according to HIF-1A gene expression into four groups, which are highly expressed (H-E), not altered (N_A), low expression, and mutated groups. Since the HIF-1A effect in HCC is the focus of our study, so we subjected the H_E and N_A groups to further analysis. The PCA of demographic data, including age, gender, ethnicity, and race between those two groups, does not show any clustering or separation, suggesting that those factors are not significantly affecting the expression profile of those patients (Fig. 2). Afterward, we analyzed the differential gene expression between the two groups and subjected the obtained results of 1852 DEGs to pathway enrichment analysis (Fig. 3). The analysis revealed the presence of 327 enriched pathways in which only 5 pathways included HIF-1A (Table 3). Metabolic reprogramming is considered one of the prominent cancer hallmarks that are impacted by the expression of our protein of interest [84]. We thus pinpointed two enriched metabolic pathways, central carbon metabolism in cancer and HIF-1 signaling pathways including 9 out of 12 genes considered as HIF-1A downstream targets (Table 4). To verify the capability of our HCC organoid to recapitulate the metabolic changes induced by the expression of HIF-1, we analyzed the relative gene expression of the nine HIF-1A targeted genes using HCC organoids. The relative gene expression of HK2, ENO2, PFKFB3, and SLC2A1 genes is consistent with their differential gene expression in which the four genes were significantly expressed in the groups with higher expression of HIF-1A (Figs. 4 and 9). In the framework, Yasuda et al. illustrated the co-expression of HIF-1A and HK2 in HCC [90]. Other studies showed downregulation of ENO2 in a neuroblastoma cell line with silencing of HIF-1A [91]. Another study on HCC demonstrated a positive feedback loop between PFKFB3 and HIF-1A that seems to contribute to resistance to the cytotoxic drug, sorafenib [92]. SLC2A1, on the other hand, is widely known as one of the key elements of glycolysis in HCC, and its expression is strongly related to the expression of HIF-1A [93].

The relative gene expressions of GCK, ENO3, and SLC2A2 were, however, inconsistent with their differential gene expression in which they were downregulated in H_E (high HIF-1A expression) samples compared to N_A (not altered) samples. However, ENO3 and SLC2A2 were significantly expressed in the organoids that expressed higher HIF-1A, and the expression of GCK was not significantly changed between the two organoid groups (Figs. 4 and 9). Our data contradict the findings of Roth et al., who suggested that binding of HIF-1A to its binding site at the GCK promoter led to activation of GCK in primary hepatocytes with the maximum response obtained in the presence of hepatocyte nuclear factor-4 (HNF-4) [94]. Our in vitro data, however, align with a study by Discher et al*.,* who reported that hypoxia augments the expression of ENO3 through the downregulation of Sp3 transcription factor, which has a repressive action on ENO3 [95]. Regarding SLC2A2, there is a lack of data that highlights the correlation between this gene and HIF-1A. However, one of the studies on pancreatic endocrine tumor illustrated the insignificant association between those genes, which might need further investigation [96].

Moreover, the differential gene expression analysis of PKM and PFKP revealed their upregulation in H_E samples, while their in vitro data demonstrated the non-significant changes between the two groups of organoids (Figs. 4 and 9). Our differential gene expression analysis data results resemble those reported by Maaty et al*.* in which PKM was downregulated in Pten/hif1a^(i)pe−/−^ luminal-C cells that have HIF-1A inactivation [97]. Although the PFKP gene was upregulated in the hypoxic conditions induced in mature adipocytes, it is not affected by the inhibition of HIF-1A specifically. This supports our RT-PCR data suggesting the role of other transcription factors rather than HIF-1A in its regulation during hypoxia [98].

Furthermore, the nine HIF-1A targeted genes are involved in two metabolic pathways: central carbon metabolism in cancer and HIF-1 signaling pathways (Table 3); these pathways are related to elevated glucose consumption and lactate production by increasing the rate of glycolysis to support the rapid growth and expansion of cancer [99, 100]. In our study, biochemical assays applied to evaluate the glycolytic activity of the two organoid groups validated the finding that the metabolic process was directed toward glycolysis in the groups with higher expression of HIF-1A. Our results showed increased glucose consumption, intracellular pyruvate, extracellular lactate with elevated gene expression of LDHA, and extracellular lactate to pyruvate ratio in HCC organoids in the hypoxic microenvironment (Fig. 10). All these data point to a glycolytic activity, which is considered one of the cancer hallmarks under the influence of HIF-1A [25, 101–104].

The concentration of NADPH decreased in HCC organoid expressing HIF-1A. Nicotinamide adenine dinucleotide phosphate oxidase 4 (NOX4) enzyme, which converts NADPH into NADP + , is one of the HIF-1A targeted genes that increase under hypoxia in HCC [105, 106]. Although the amount of ATP produced from OXPHOS is more than glycolysis for each glucose molecule [107], our data showed no significant difference in the ATP produced in both groups of organoids. This may reflect the decline in ATP cellular utilization because of suppressed Na-K-ATPase activity (consuming 20–70% of ATP) during hypoxia [108–110] or the rapid ATP generation rate from glycolysis compared to OXPHOS [111]. Notably, the Kaplan–Meier survival analysis illustrated the clinical significance of HIF-1A and its downstream genes in HCC patients, as the group with elevated expression of HIF-1A, HK2, ENO2, SLC2A1, PKM, and PFKP, and lower expression of GCK and SLC2A2 was associated with poor prognosis and low survival rate when compared to the counterpart group of low expression (Fig. 5). These data suggest the important role of these genes in developing unfavorable implications in HCC patients.

## Conclusions

In conclusion, this study highlighted four of the nine targeted genes of HIF-1A, HK2, ENO2, PFKFB3, and SLC2A1, which are involved in the metabolic pathways. Those genes can present therapeutic targets for the treatment of HCC patients, especially those with hypoxic microenvironment within their solid tumors, which may contribute to chemotherapeutic resistance, poor prognosis, and low survival rate.

We also demonstrated that HCC-mixed organoids comprising heterogeneous populations of cells can grow spontaneously and generate a hypoxic microenvironment with a stabilized expression of HIF-A within the organoids without exposure to CoCl_2_. These organoids can thus be employed as an in vitro model for studying the effect of HIF-1A on cancer-related properties and for high-throughput screening of chemotherapeutic drugs.

## Supplementary Information

Below is the link to the electronic supplementary material.

Supplementary file1 (XLSX 329 KB) Online Resources .xlsx: it contains the following sheets: Sheet 1: it contains article title, journal name, author names, affiliation and e-mail address of the corresponding author. Online Resource 1: It contains the retrieved 372 samples CBioPortal. The samples were labeled with one of five groups according to HIF-1A expression as follows: high HIF-1A expression, low HIF-1A expression, mutated HIF-1A gene, not profiled, and unaltered expression. Online Resource 2: Samples that were retrieved via CBioPortal. The samples were divided into two labels according to HIF-1A expression: Highly expressed and Not Altered. The table includes the demographic data for each patient including age, ethnicity, gender, and race. Online Resource 3: The identified 1852 DEGs between H_E and N_A groups were with a cutoff of |log2FC| > 2 and adjusted P-value < 0.01. Online Resource 4: It includes 582 proteins of the protein-protein interaction network with their degree values. Online Resource 5: A total of 327 enriched pathways were identified utilizing G: Profiler with False Discovery Rate (FDR) < 0.01

Supplementary file2 (PDF 408 KB) S_Figure 1_Protein Protein Interaction Network.pdf: A protein-protein interaction network with 1136 proteins connected by 1713 edges was constructed. Nodes with zero degrees were excluded from the network diagram

## Data Availability

The dataset analyzed during the current study is available at the Cancer Genome Atlas (TCGA) (project ID: TCGA-LIHC), https://portal.gdc.cancer.gov/projects/TCGA-LIHC All codes, data for reproducing the Deseq2 analysis results, and visualization figures can be found in the Github repository. See Code https://github.com/ComputationalBiologyLab/Target-genes-of-HIF-1A-in-HCC-patients.git. All codes, data for reproducing the Deseq2 analysis results, and visualization figures can be found in the Github repository. See Code https://github.com/ComputationalBiologyLab/Target-genes-of-HIF-1A-in-HCC-patients.git

## Abbreviations

HCC: Hepatocellular carcinoma
HIF-1A: Hypoxia-inducible factor-1A
2D: 2 Dimensional
ECM: Extracellular matrix
TME: Tumor microenvironment
HIFs: Hypoxia-inducible factors
PHDs: Prolyl hydroxylase domain-containing proteins
VHL: Von Hippel-Lindau tumor suppressor protein
HRE: Hypoxia-responsive element
TCA: Tricarboxylic acid cycle
OXPHOS: Oxidative phosphorylation
ENO1: Enolase 1
LDHA: Lactate dehydrogenase A chain
PFKFB3: 6-Phosphofructo-2-kinase/fructose-2,6-bisphosphate 3
HK1 and HK2: Hexokinases
GAPDH: Glyceraldehyde 3-phosphate dehydrogenase
PFKL: Phosphofructokinase-liver type
ALD-A and ALD-C: Aldolases
GLUT1 and GLUT3: Glucose transporter proteins
PDK1: Pyruvate dehydrogenase kinase 1
Acetyl-CoA: Acetyl coenzyme A
GSEA: Gene set enrichment analysis
PGCM: Phosphoglucomutase
VEGF: Vascular endothelial growth factor
TGF-*β*: Transforming growth factor-*β*
MMP9: Matrix metallopeptidase 9
TNF-*α*: Tumor necrotic factor-*α*
LIHC: Liver hepatocellular carcinoma
TCGA: Cancer Genome Atlas
H_E: Highly expressed
N_A: Not altered
PPI: Protein–protein interaction
DEGs: Differentially expressed genes
PCA: Principle component analysis
FDR: False discovery rate
*P*_adj_: Adjusted *P* value
Log2FC: Log2 fold change
hBM-MSCs: Human bone marrow-derived mesenchymal stem cells
DMEM: Dulbecco's modified Eagle medium
FBS: Fetal bovine serum
HUVEC: Human umbilical vein endothelial cells
b-FGF: Basic fibroblast growth factor
EGF: Epidermal growth factor
PRP: Platelets-rich plasma
CCM: Complete culture medium
PBS: Phosphate buffer saline
DMSO: Dimethyl sulfoxide
OD: Optical density
PI: Propidium iodide
BSA: Bovine serum albumin
LDH: Lactate dehydrogenase
HK2: Hexokinase 2
PKM: Pyruvate kinase M
GCK: Glucokinase
ENO2: Enolase 2
ENO3: Enolase 3
PFKP: Phosphofructokinase
SLC2A1: Solute carrier family 2 member 1
SLC2A2: Solute carrier family 2 member 2
ROS: Reactive oxygen species
HNF-4: Hepatocyte nuclear factor-4
NOX4: Nicotinamide adenine dinucleotide phosphate oxidase 4

## Footnotes

1
https://portal.gdc.cancer.gov/projects/TCGA-LIHC

2 https://bioconductor.org/packages/release/bioc/html/GenomicDataCommons.html

3 https://www.cbioportal.org/

4 https://bioconductor.org/packages/release/bioc/html/DESeq2.html

5 https://ggplot2.tidyverse.org/

6
https://string-db.org/

7 https://apps.cytoscape.org/apps/networkanalyzer

8 https://biit.cs.ut.ee/gprofiler

9 https://cran.r-project.org/web/packages/survival/index.html

10 https://cran.r-project.org/web/packages/survminer/index.html

11 https://cran.r-project.org/web/packages/cowplot/vignettes/introduction.html
