# Supplementary material for: Development of hepatocellular carcinoma organoid model recapitulating HIF-1A metabolic signature
Source: Clin Exp Med. 2024 Nov 21;25(1):9. doi: 10.1007/s10238-024-01521-x (PMC11579110; doi:10.1007/s10238-024-01521-x)
Supplement: Supplementary file 2 — Supplementary file2 (PDF 408 KB) S_Figure 1_Protein Protein Interaction Network.pdf: A protein-protein interaction network with 1136 proteins connected by 1713 edges was constructed. Nodes with zero degrees were excluded from the network diagram [file 10238_2024_1521_MOESM2_ESM.pdf]

Development of Hepatocellular Carcinoma Organoid Model Recapitulating HIF-1A Metabolic Signature

Mennatallah A. Khedr[1+], Zainab Mohamed[2+], Azza M. El-Derby[1+], Malak M. Soliman[3], Amira Abdel Fattah Edris[4,5], Eman Badr[2, 6*], Nagwa El-Badri[1,2*]

[1]Center of Excellence for Stem Cells and Regenerative Medicine, Zewail City of Science and Technology, Giza, 12578, Egypt

[2]University of Science and Technology, Zewail City of Science and Technology, Giza, 12578, Egypt

[3]School of Information Technology and Computer Science, Nile University, 12677, Giza, Egypt

[4] Department of Pediatrics, Cairo University, Cairo, 11956, Egypt

[5] Rofayda Medical Center, Giza, 3240020, Egypt

[6] Faculty of Computers and Artificial Intelligence, Cairo University, Giza, 12613, Egypt

* Correspondence to emostafa@zewailcity.edu.eg and nelbadri@zewailcity.edu.eg

+ These authors contributed equally to this work.

Clinical and Experimental Medicine

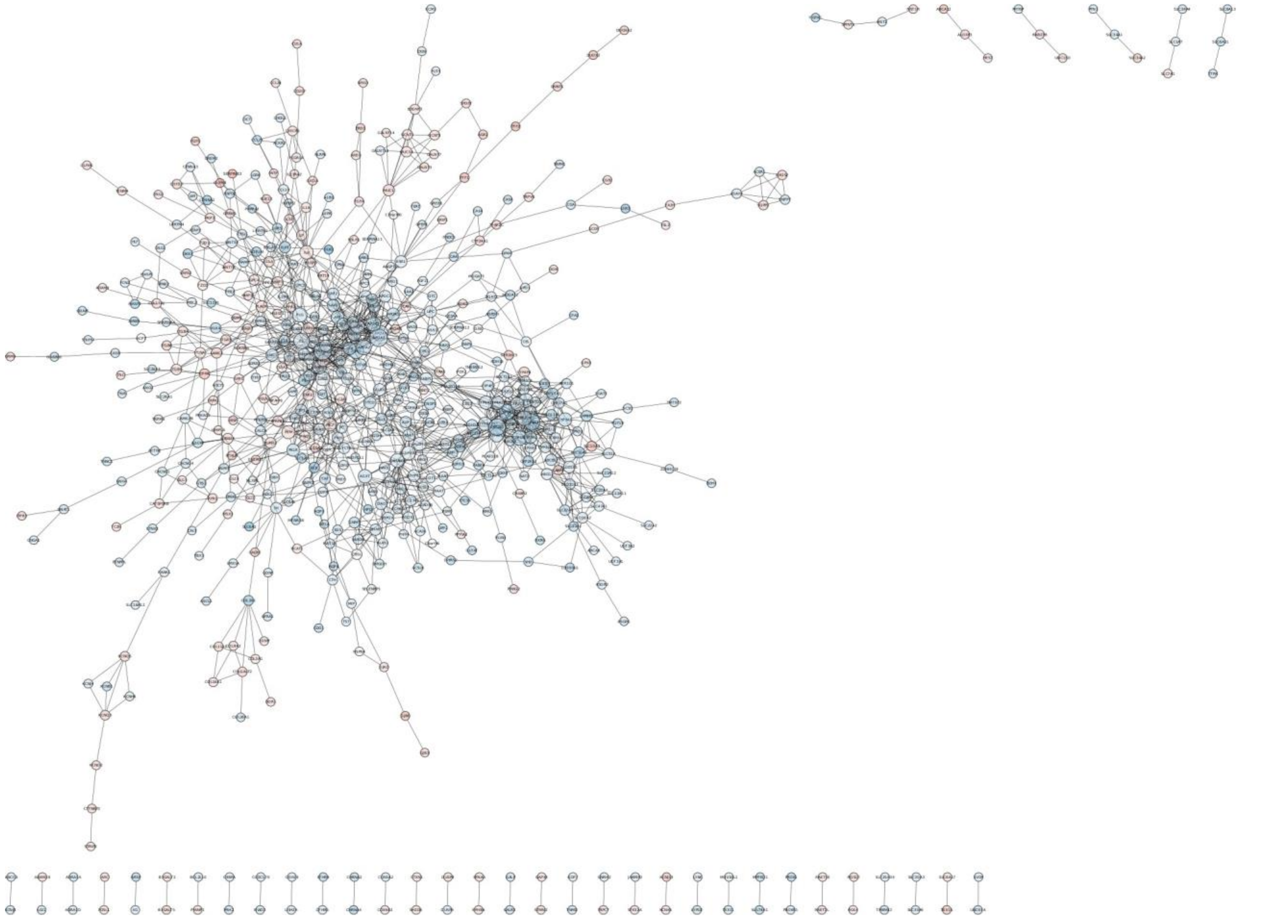

S_Figure 1: Protein-Protein Interaction Network
